# Early B-cell factors 2 and 3 (EBF2/3) regulate early migration of Cajal–Retzius cells from the cortical hem

**DOI:** 10.1016/j.ydbio.2012.02.034

**Published:** 2012-05-01

**Authors:** Francesca Chiara, Aurora Badaloni, Laura Croci, Mason L. Yeh, Anna Cariboni, Anna Hoerder-Suabedissen, G. Giacomo Consalez, Britta Eickholt, Tomomi Shimogori, John G. Parnavelas, Sonja Rakić

**Affiliations:** a Department of Cell and Developmental Biology, University College London, UK; b San Raffaele Scientific Institute, 20132 Milan, Italy; c MRC Centre for Developmental Neurobiology, King's College London, UK; d Department of Physiology, Anatomy and Genetics, University of Oxford, UK; e RIKEN Brain Science Institute, Saitama, Japan

**Keywords:** Corticogenesis, Migration, Reelin, Transcription factor

## Abstract

Cajal–Retzius (CR) cells play a crucial role in the formation of the cerebral cortex, yet the molecules that control their development are largely unknown. Here, we show that *Ebf* transcription factors are expressed in forebrain signalling centres—the septum, cortical hem and the pallial–subpallial boundary—known to generate CR cells. We identified *Ebf2*, through fate mapping studies, as a novel marker for cortical hem- and septum-derived CR cells. Loss of *Ebf2* in vivo causes a transient decrease in CR cell numbers on the cortical surface due to a migratory defect in the cortical hem, and is accompanied by upregulation of *Ebf3* in this and other forebrain territories that produce CR cells, without affecting proper cortical lamination. Accordingly, using in vitro preparations, we demonstrated that both *Ebf2* and *Ebf3*, singly or together, control the migration of CR cells arising in the cortical hem. These findings provide evidence that *Ebfs* directly regulate CR cell development.

## Highlights

*► Ebf2* is a novel marker for hem- and septum-derived Cajal–Retzius cells. *► Ebf2* mutant shows a transient migratory defect of hem-derived Cajal–Retzius cells. *► Ebf3* is expressed in similar territories as *Ebf2* and rescues *Ebf2* mutant phenotype. ► Both *Ebf2* and *Ebf3* are required for Cajal–Retzius cell migration.

## Introduction

The cerebral cortex is a highly organized structure, subdivided in radial (layers) and tangential (areas) domains, and involved in complex functions such as cognition, sensory processing and motor control. There is emerging evidence that Cajal–Retzius (CR) cells, the earliest neurons generated in the developing cortex, play a crucial role in both its laminar (Cooper, 2008; Rice and Curran, 2001; Soriano and Del Rio, 2005; Tissir and Goffinet, 2003) and areal (Bielle et al., 2005; Griveau et al., 2010; Meyer et al., 2004) specification.

Cajal–Retzius cells arise in a number of forebrain signalling centres, such as the cortical hem (CH) (Garcia-Moreno et al., 2007; Takiguchi-Hayashi et al., 2004), septum/retrobulbar area and pallial–subpallial boundary (PSPB; also known as the anti-hem) (Bielle et al., 2005) and, following tangential migration, populate the entire cortical surface (Bielle et al., 2005; Yoshida et al., 2006). Recently, the thalamic eminence and choroid plexus (ChP) have also been proposed as sites of origin for CR cells (Abellan et al., 2010; Imayoshi et al., 2008; Tissir et al., 2009). The most widely accepted function of CR cells is in cortical lamination; these cells secrete Reelin (D'Arcangelo et al., 1995; Ogawa et al., 1995; Rice and Curran, 2001) to orchestrate neuronal migration in the typical “inside-out” pattern (Caviness, 1982). However, studies that demonstrated multiple origins of CR cells (Bielle et al., 2005; Garcia-Moreno et al., 2007) support the hypothesis that they could also provide information important for correct cortical areal patterning.

A number of transcription factors have been implicated in the control of CR cell differentiation, migration and survival, but the underlying mechanisms are poorly understood. Early observations pointed to a role of genes such as *Tbr1*, *Pax6*, *Emx1* and *Emx2* (Hevner et al., 2001; Mallamaci et al., 2000; Muzio and Mallamaci, 2003), as well as *Foxg1,* a repressor of CR cell fate in vivo (Hanashima et al., 2004) and in vitro (Hanashima et al., 2007; Shen et al., 2006). Factors related to particular CR cell subpopulations have also been identified: *p73* (Meyer et al., 2002), *p21* (Siegenthaler and Miller, 2008) and *Zic1*-*3* (Inoue et al., 2008) for septum- and CH-derived cells, *Dbx1* for septum- and PSPB-derived cells (Bielle et al., 2005), and *Lhx5* and *Er81* for cells of ChP and septal origin, respectively (Imayoshi et al., 2008; Zimmer et al., 2010). Moreover, recent studies have revealed a novel role for *LIM*-*homeobox genes* in maintaining CR cell development in primates, birds and rodents (Abellan et al., 2010). On the other hand, cortical meninges expressing the chemokine Cxcl12 (or SDF-1) have been found to act as substrate for CH-derived CR cells as well as to provide chemoattractant signals during their migration (Borrell and Marin, 2006).

Recent studies have shown that *Collier/Olf/Ebfs* (COE) transcription factors are transiently expressed in the cerebral cortex (Garel et al., 1997) and, specifically, *Ebf2* and *Ebf3* are expressed in CR cells during corticogenesis (Chowdhury et al., 2010; Hanashima et al., 2007; Yamazaki et al., 2004). *Ebf* genes encode helix–loop–helix transcription factors highly conserved in evolution (Dubois and Vincent, 2001; Malgaretti et al., 1997; Wang et al., 1997). They are implicated in various aspects of neural development, including neuronal differentiation (Dubois et al., 1998; Pozzoli et al., 2001), migration (Garcia-Dominguez et al., 2003; Garel et al., 2000) and axon fasciculation and guidance (Garel et al., 1997, 1999, 2002; Malgaretti et al., 1997; Prasad et al., 1998; Wang et al., 1997). One member of this family, *Ebf2*, plays an important role in neuroendocrine, olfactory, skeletal and peripheral nerve development (Corradi et al., 2003; Giacomini et al., 2011; Kieslinger et al., 2005; Wang et al., 2004). In cerebellum, *Ebf2* is involved in patterning of the cortex (Chung et al., 2008; Croci et al., 2006) and Purkinje cell survival (Croci et al., 2011). In the present study, we identified *Ebf2* as a novel marker of CH- and septum-derived CR cells. Loss of *Ebf2* in vivo causes a transient decrease in numbers of CR cells at the cortical surface, accompanied by upregulation of *Ebf3* in the CH and PSPB and increased production of CR cells in the CH, with no defect in cortical lamination. Furthermore, using in vitro preparations, we discovered that *Ebf2* and *Ebf3*, singly or cooperatively, directly control migration of CR cells emanating from the CH.

## Materials and methods

### Animals

All experimental procedures were conducted in accordance with the UK Animals (Scientific Procedures) Act 1986 and institutional guidelines.

**Supplementary Fig. 1:**
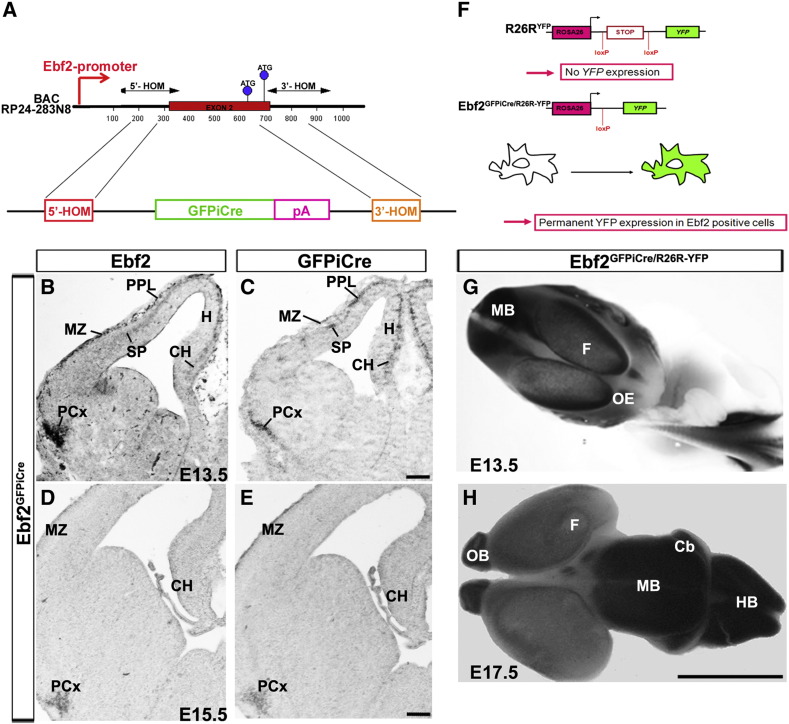
Validation of the *Ebf2*^*GFPiCre*^ transgenic mouse.

The mouse lines *Ebf2*^*+/−*^ (Croci et al., 2006)*, Gt (ROSA)26Sor*^*tm1(EYFP) Cos*^ (*R26R*^*YFP*^; Jackson Laboratories) and *Ebf2*^*GFPiCre*^ (Figs. S1A–H; Badaloni A., Chiara F. and Consalez G.G., unpublished) were used in this study. Embryos were fixed overnight in 4% paraformaldehyde (PFA) in phosphate buffer saline (PBS). Mice of postnatal ages were anesthetised by isofluothane and transcardially perfused with PFA. For genotyping, DNA from tissue biopsies was analysed by polymerase chain reaction using PCR Master Mix (Promega) and specific primers (Table S1). *Ebf2*^*+/−*^ did not show any phenotype when compared to *Ebf2*^*+/+*^ littermates (Croci et al., 2006; Chiara F. and Rakic S., unpublished observations). In this study *Ebf2*^*+/+*^ were used as wild type (w-t) controls and compared to mutants or transgenic mice of the same litter.

### RNA extraction and RT-PCR

RNA was extracted from cells using a commercially available kit from Qiagen. First strand DNA was synthesised from RNA using the kit SuperscriptII RT (Invitrogen). Sequences of the primers used are in the Supplementary data (Table S2).

### In situ hybridization

In situ hybridization experiments were performed as described by C. Ragsdale (http://ragslab.bsd.uchicago.edu/index.html, University of Chicago, Chicago, USA). One- and two-color in situ hybridization was carried out on embryonic brains, sectioned by freezing microtome (40 μm). Digoxygenin- and fluorescein-labelled riboprobes (Roche) were transcribed from plasmids containing *Ebf1, Ebf2, Ebf3, Reelin* (Croci et al., 2006), *Dbx1*, *Wnt3a, Cxcr4 and Sdf1* (Stumm et al., 2003) mouse cDNAs.

### Cell culture and DNA transfection

COS7 (American Type Culture Collection) and GN11 cell lines (kindly provided by Prof. R. Maggi, Universitá Statale di Milano, Milan, Italy) were maintained in Dulbecco Modified Eagle's Media DMEM (Invitrogen) supplemented with 10% foetal bovine serum (FBS; Invitrogen). Cells were transfected with Lipofectamine 2000 (Invitrogen). List of plasmids used can be found in the Supplementary data (Table S3). For Ebf-flag and shRNA specifications refer to Croci et al. (2011).

### Immunohistochemistry

Embryonic brains were cryoprotected in 30% sucrose in PBS, frozen in Tissue Tek O.C.T. (Sakura Finetek) and sectioned using a Cryostat (20 μm; Bright Instruments). Postnatal brains were frozen on dry ice and sectioned by freezing microtome (40 μm; Leica SM2010 R). Immunohistochemistry was performed as previously described (Rakic et al., 2009). List of antibodies used can be found in the Supplementary data (Tables S4 and S5).

### Nuclear staining

Sections were stained either with 0.025% thionin solution (Nissl staining; Sigma) or with 2.5 μg/mL bisbenzimide (Sigma).

### Cytochrome oxidase histochemistry

Cytochrome oxidase histochemistry has been preformed according to the published protocols of Hoerder-Suabedissen et al. (2008).

### Western blotting

COS7 cells were harvested 48 h after transfection and lysed in RIPA buffer containing protease and phosphatase inhibitors. Protein concentration was determined by Bradford assay. Lysates were separated on an SDS-polyacrylamide gel at 10%. Proteins were transferred onto a nitrocellulose membrane (Amersham-GE Healthcare). Enhanced Chemiluminescence reagent (ECL, Amersham) was used, according to manufacturer's instructions. The reaction was detected using a GEL-DOC detection machine (Bio-Rad). List of antibodies used can be found in the Supplementary data (Table S4 and S5).

### Migration assays

Chemotaxis assays were performed using a 48-well Boyden's chamber (NeuroProbe) as described previously (Cariboni et al., 2007). Migrated cells were counted in three random fields per well.

### Dissociated cell cultures

Cortical hems were dissected from E11.5 mouse brains and incubated in 0.05% trypsin with 100 μg/mL DNaseI in Neurobasal medium (Invitrogen) at 37 °C for 15 min. Trypsinization was quenched and cells resuspended in growth media (Neurobasal medium, 2 mM l-glutamine, 50 units/mL penicillin, and 50 μg/mL streptomycin, with 1:50 dilution of B27; all reagents from Invitrogen). Cells were nucleofected using the Mouse Neuron Nucleofection Kit according to the manufacturer's instructions (Program O-05; LONZA).

### Stripe assay

Silicon matrices and equipment for the stripe assay and experiments were performed as described previously (Eickholt et al., 1999). Coverslips, coated with poly-l-lysine (PLL, Invitrogen), where placed on top of silicon matrices. Laminin (LN, 40 μg/mL, Invitrogen) was injected into the matrices to create alternate stripes and incubated at 37 °C for 2 h. Coverslips were detached from the matrices and nucleofected cells (300,000/mL) were plated onto stripe/coated coverslips and incubated for 72 h. Cells were counted in each stripe, and the number adjusted to the stripe area measured with ImageJ (NIH).

### Digital image capture and analysis

Brightfield images were taken using a Leica DM microscope with DC500 digital camera. Immunofluorescent samples were photographed using Leica TCS SP1 confocal microscope. All images were processed using Photoshop CS3 software (Adobe).

### Cell counts

Cell counts were performed using Metamorph (Molecular Devices) software. Only cells showing visible neurites or nuclei were considered for cytoplasmic staining and nuclear staining, respectively. At E13.5, all cells in the pallial area were counted; at E16.5 or postnatally, the number of cells was counted in boxes that were placed over pallial regions matching control and mutant sections.

### Statistical analysis

For all experiments, the mean of at least three independent samples was calculated. Data are expressed as mean ± s.e.m. Statistical significance was determined by using a paired *t*-test. Statistical significance between group means was tested by one-way ANOVA followed by Bonferroni's post hoc test. All statistical analyses were performed by Prism4 software (GraphPad).

## Results

### Ebf2 is expressed in post**-**mitotic neurons in the developing mouse forebrain

**Fig. 1:**
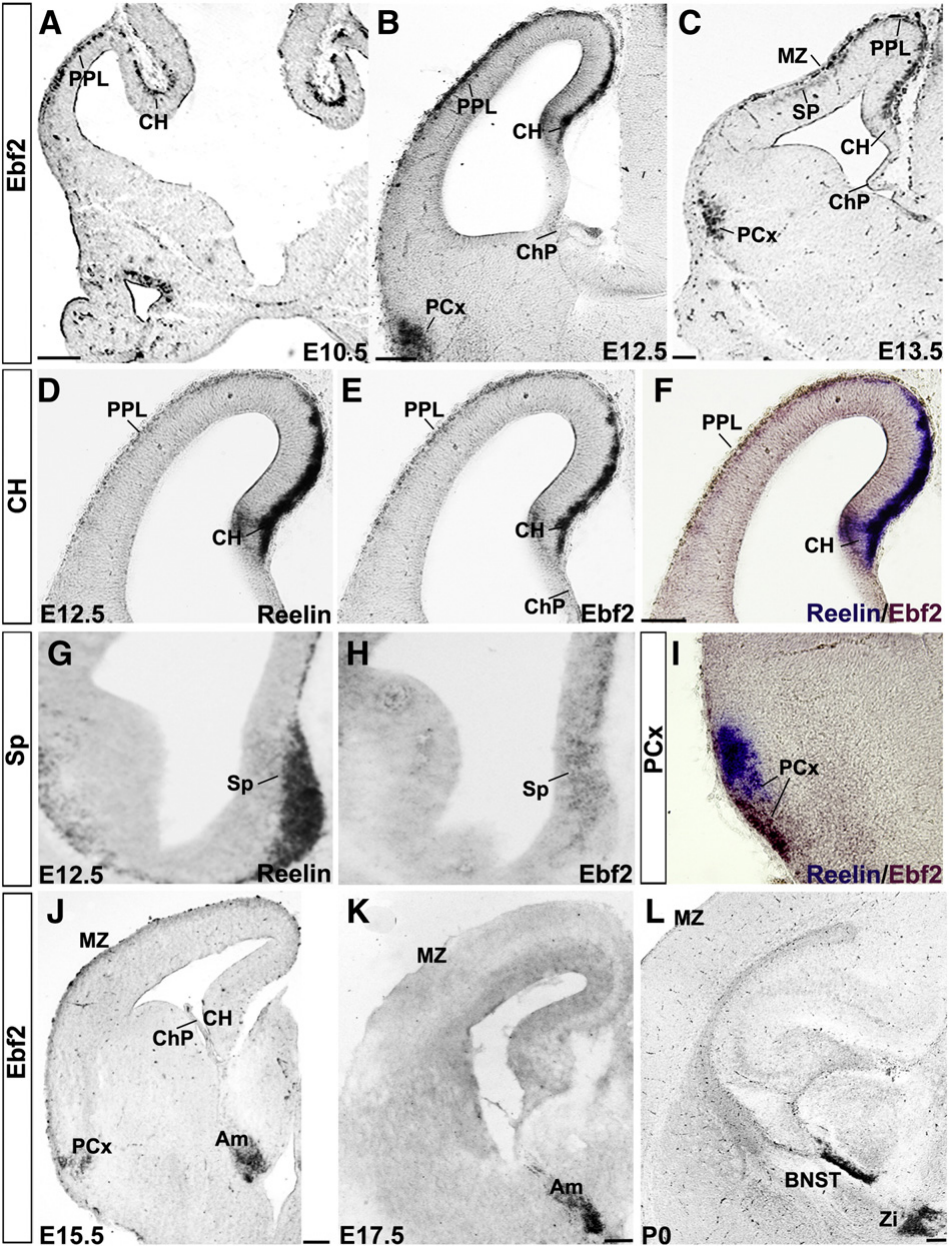
*Ebf2* expression during forebrain development. Between E10.5 and E13.5, *Ebf2* is detected in the PPL, CH, PCx (A–C). *Reelin* and *Ebf2* expression match in the PPL and CH (D–F). Moreover, *Reelin* and *Ebf2* are expressed in the septum. *Reelin* and *Ebf2* do not co-localise in the PCx (I). *Ebf2* is not expressed in the ChP (B, C, E and J). *Ebf2* is downregulated in pallial regions from E15.5. However, expression persists in the PCx and Am (J, K). Postnatally *Ebf2* is localised in Zi and BNST (L). Am: amygdala, BNST: bed nucleus of stria terminalis, CH: cortical hem, ChP: choroid plexus, MZ: marginal zone, Sp: septum, SP: subplate, PCx: piriform cortex, PPL: preplate, Zi: zona incerta. Scale bars: (A–I) 100 μm, (J–L) 200 μm.

Although *Ebf2* is transiently expressed in post-mitotic forebrain neurons (Garel et al., 1997), its role in the developing cerebral cortex has never been established. In the present work, we first describe in detail the pattern of *Ebf2* expression during mouse forebrain development. Forebrain sections, ranging in age from E10.5 to newborn, were analysed using in situ hybridization. *Ebf2* expression was first detected in the CH and preplate (PPL) at E10.5 (Fig. 1A). At E12.5, its expression was extended to piriform cortex (PCx), which is part of the mantle zone of the ventral pallium (Fig. 1B), and septum (Fig. 1H). At this age, the expression of *Reelin*, a marker of CR cells, matched the expression of *Ebf2* in the PPL, CH and septum (Figs. 1D–H). Interestingly, *Ebf2* expression did not coincide with *Reelin* expression in the PCx (Fig. 1I); thus, we hypothesised that *Ebf2*^*+*^ signal in this area does not represent CR cells, but rather fibers of the lateral olfactory tract (Corradi et al., 2003). According to Imayoshi et al. (2008), the ChP gives rise to a subpopulation of *Lhx5*^+^ and *Reelin*^+^ CR cells that eventually migrate into the PCx. However, in agreement with our hypothesis, *Ebf2* was not expressed in the ChP (Figs. 1B,C,E,J). At E13.5, *Ebf2* was still expressed in the septum (not shown), CH, marginal zone (MZ) and subplate (SP) layers, both derivatives of PPL and PCx (Fig. 1C). A general downregulation of the *Ebf2* gene occurred in the MZ and CH at E15.5, whereas its expression in the PCx and subcortical nuclei persisted (Figs. 1J,K). At P0, *Ebf2* expression was detected only in the zona incerta and bed nucleus of stria terminalis (Fig. 1L).

### Ebf2 is specifically expressed in CR cells

**Fig. 2:**
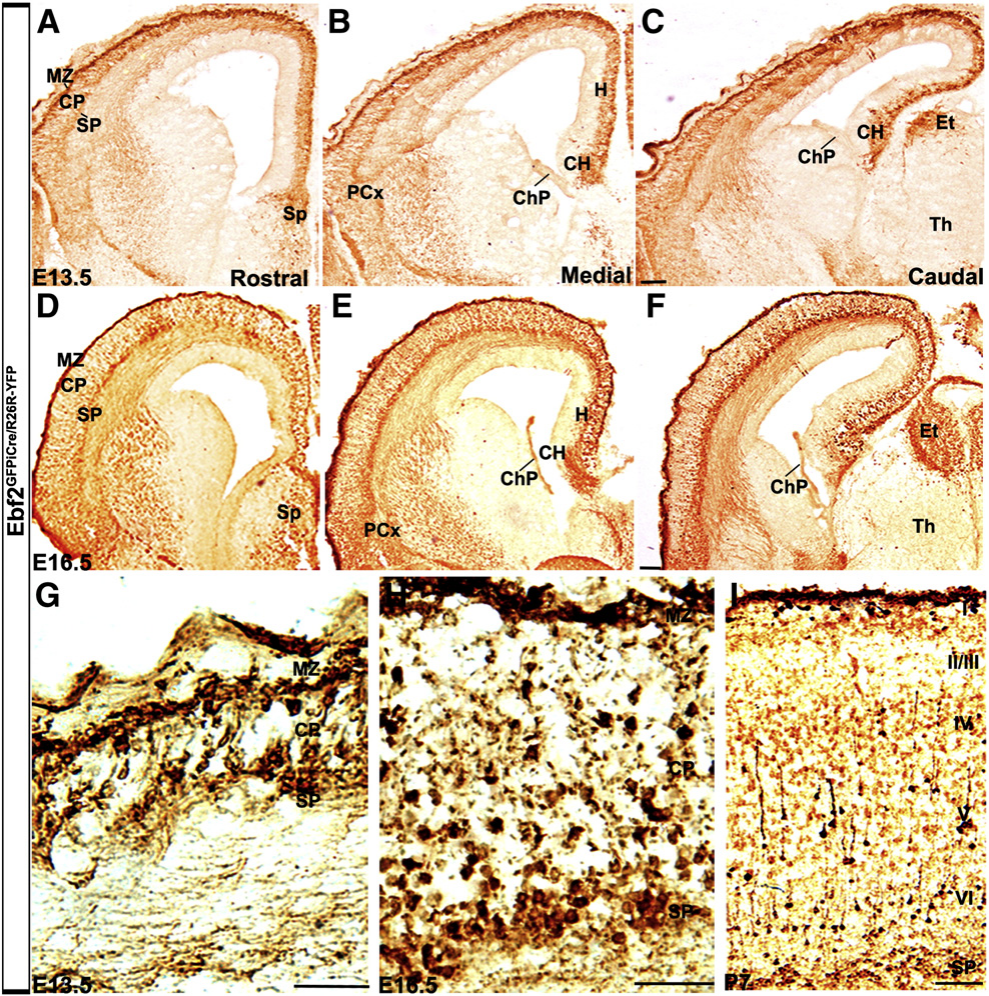
Fate mapping of *Ebf2*^+^ cells in *Ebf2*^*GFPiCre/R26R-YFP*^. At E13.5 and E16.5, the transgene is expressed in the MZ, SP, CH, H, PCx, Sp and Et, but not in Th and ChP (A–F). Observation at higher magnification reveals that *Ebf2* is also expressed in SP cells and neurons in the CP (G, H). At P7, YFP cells are in layer I, layer V and at the bottom of layer VI (SP cells, I). CH: cortical hem, ChP: choroid plexus, CP: cortical plate, Et: epithalamus, H: hippocampus, MZ: marginal zone, SP: subplate, Sp: septum, PCx: piriform cortex, Th: thalamus. Scale bars: (A–F, I) 100 μm, (G) 25 μm, (H) 50 μm.

**Fig. 3:**
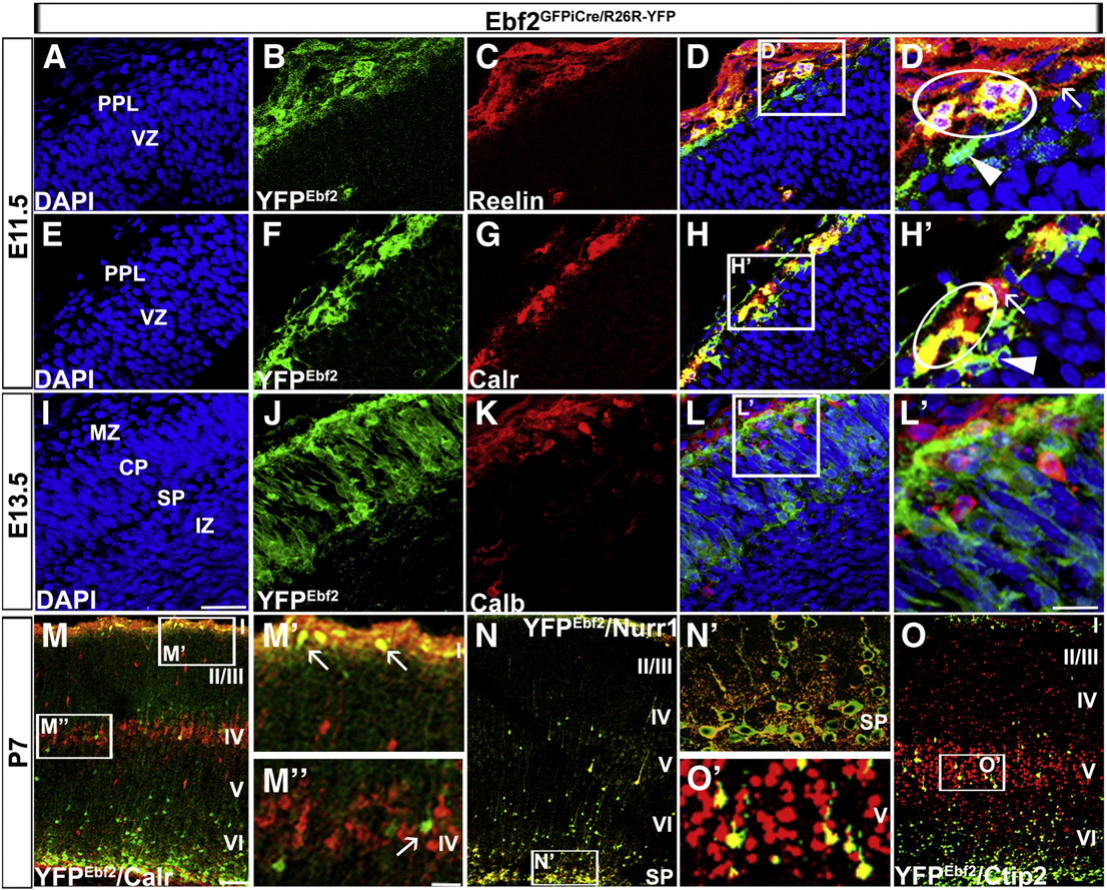
*Ebf2* is expressed in CR, SP and pyramidal cell subpopulations, but not in interneurons. At E11.5, YFP^*Ebf2*^ expression coincided with that of Reelin (A–D′; circle in D′ highlights double positive cells, arrowhead points to YFP^*Ebf2*+^ cells only and arrow points to Reelin^+^ cells only) and Calr (E–H′; circle in H′ shows double positive cells, arrowhead points to green cells only, and arrow points to Calr^+^ cells only). At E13.5, migrating cortical interneurons in the MZ (I–L′) were not YFP^*Ebf2*+^ (L′). At P7, YFP^*Ebf2*^ was expressed in CR cells in layer I (M, M′; arrow), but not in interneurons (M″; arrow points to YFP^*Ebf2*+^ cell in layer IV not expressing Calr). Nurr1 (N) YFP^*Ebf2 +*^ cells are also found at the bottom of layer VI (N′). YFP^*Ebf2*+^ cells were also positioned in layer IV (M, M″; arrow) and V (Ctip2^+^: O, O′). CP: cortical plate, IZ: intermediate zone, MZ: marginal zone, PPL: preplate, SP: subplate layer, VZ: ventricular zone. Scale bars: (A–L; M′, M″, N′ and O′) 50 μm, (D′, H′ and L′) 25 μm, (M, N and O) 100 μm.

We then used an *Ebf2*^*GFPiCre*^ transgenic line to perform fate-mapping studies on *Ebf2*^*+*^cells. This line was obtained through the integration of the fusion protein GFPiCre (Badaloni A., unpublished) into the first coding exon of the *Ebf2* locus in the BAC RP24-283N8 (Fig. S1A). The expression of the transgene faithfully mimicked the expression of the endogenous gene (Figs. S1B–E). In order to permanently label the lineage of *Ebf2*^*+*^ cells, the transgenic line was crossed with the reporter line *R26R*^*LacZ*^ (Figs. S1F–H). YFP^*Ebf2*^ immunohistochemistry, at E13.5 and E16.5, revealed that *Ebf2* was expressed along the rostro-caudal axis in forebrain structures such as neocortex, CH, septum, hippocampus and PCx (Figs. 2A–F). Moreover, YFP^*Ebf2*^-expressing cells in the neocortex were detected in the MZ, cortical plate (CP) and SP (Figs. 2G,H). In the diencephalon, the transgene was expressed in the epithalamus (Figs. 2C,F). No expression was detected in the ChP and thalamic nuclei or axons arising from this area (Figs. 2B,C,E,F). At P7, YFP^*Ebf2*^ cells in the cerebral cortex were positioned in layers I (former PPL/MZ), IV and V (Figs. 2I, 3M,O), and in the SP (Figs. 2I,3N,N′).

We next looked for cell-specific expression of YFP^*Ebf2*^ in the cortex. At E11.5, the PPL is almost entirely populated by migrating CR cells, and YFP^*Ebf2*^ expression coincided with that of Reelin (Figs. 3A–D′; 30% of YFP^*Ebf2*^ cells were Reelin^+^, data not shown) and Calretinin (Calr; Figs. 3E–H′, 39.8% of YFP^*Ebf2*^ cells were Calr^+^, data not shown), another marker of CR cells. Earlier studies have shown that Reelin expression in the CH appears after E11.5 (Hanashima et al., 2007; Meyer et al., 2002). Also, it has been shown that the polyclonal anti-Calr antibody, produced by Swant and used in the present experiments, does not recognise a subpopulation of Calr^+^ CR cells deriving from the septum (Bielle et al., 2005). Thus, it is likely that YFP^*Ebf2*+^/Reelin^+^ cells were septum- (Griveau et al., 2010), while YFP^*Ebf2 +*^/Calr^+^ were CH-derived CR cells. On the other hand, YFP^*Ebf2*+^/Reelin^−^ (Fig. 3D′) and YFP^*Ebf2*+^/Calr^−^ (Fig. 3H′) cells were probably CR cells that originated from the CH and septum, respectively. Finally, YFP^*Ebf2*−^/Reelin^+^ (Fig. 3D′) as well as YFP^*Ebf2*−^/Calr^+^ (Fig. 3H′) cells could be CR cells that emanated from the PSPB, where *Ebf2* was not expressed. A percentage of YFP^*Ebf2*+^/Calr^+^ cells detected after E12.5 were SP cells, but these cells were difficult to discriminate from CR cells at early developmental stages; they were found in the SP layer after the splitting of the PPL (Figs. 2G–I). At E13.5, migrating interneurons were also positioned in the MZ (Figs. 3I–L′) and specifically labelled with Calbindin (Calb) antibody. Calbindin immunoreactivity was not detected in YFP^*Ebf2*+^ cells, suggesting that *Ebf2* was not expressed in migrating interneurons (Fig. 3L′). At P7, YFP^*Ebf2*^ was expressed in CR cells, labelled with Calr antibody in layer I (Figs. 3M,M′). However, a subpopulation of interneurons, expressing Calr postnatally, did not contain YFP^*Ebf2*^ (Fig. 3M″). YFP^*Ebf2*+^ cells expressed Nuclear receptor related 1 (Nurr1) (Figs. 3N,N′), a postnatal marker for SP cells, confirming our previous immunohistochemical observations (Figs. 2G–I). Moreover, a small number of YFP^*Ebf2*+^ cells, seen in the lower parts of developing CP at E13.5 and E16.5 (Figs. 2G,H), were positioned in layers V (Ctip2^+^; Figs. 3O,O′) and IV (Cux1^+^; data not shown) postnatally.

Our in situ hybridization and fate mapping studies demonstrated that *Ebf2* was present mainly in the septum- and hem-derived CR cell subpopulations, but also in pyramidal cells of layers IV and V, as well as in SP cells. Previous reports have shown that *Ebf2*^*−/−*^ mice display multiple phenotypes, including cell specification and migration defects in the cerebellum and olfactory system (Corradi et al., 2003; Croci et al., 2006), prompting us to investigate the role of this gene in forebrain development.

### Cortical hem**-**derived CR cell development is affected in Ebf2^−/−^ mice

**Fig. 4:**
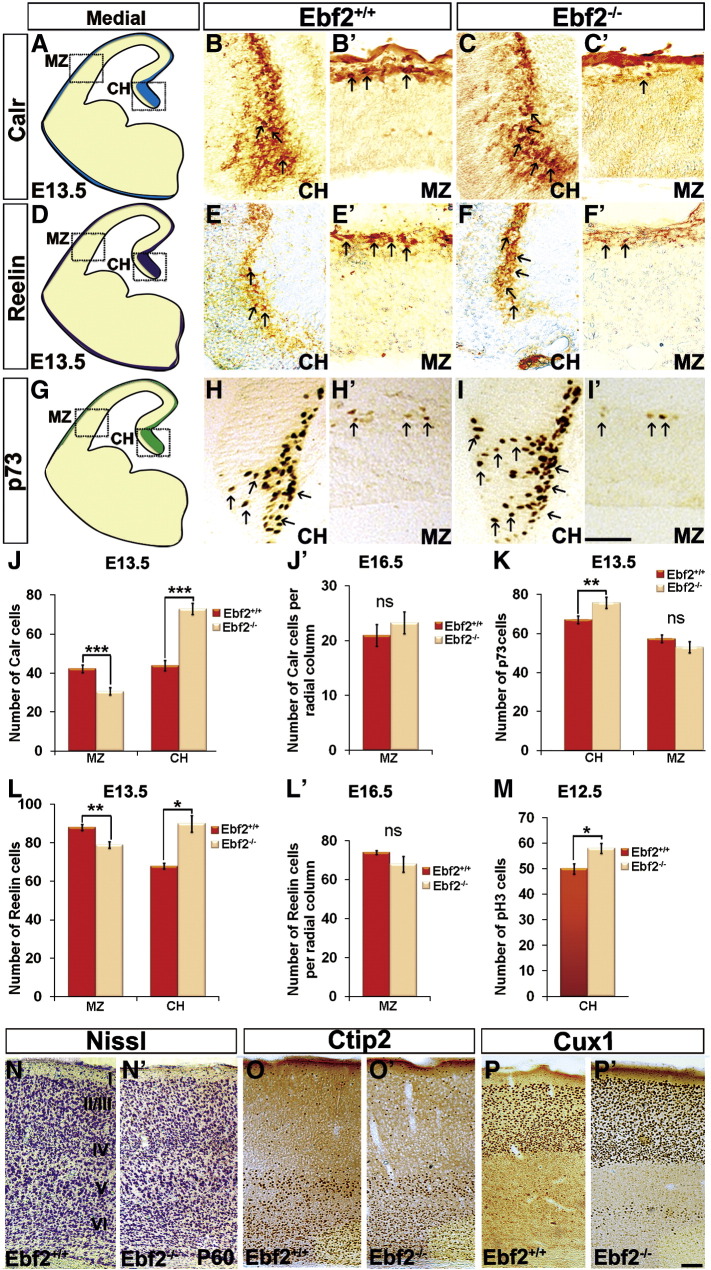
CR cell migration is temporally delayed, but does not lead to major defects in *Ebf2*^*−/−*^ cortices. Coronal medial sections were considered as shown in the schemes (A, D and G). At E13.5, expression of Calr, Reelin and p73 is shown in the CH (B and C, Calr; E and F, Reelin; H and I, p73) and MZ (B′ and C′, Calr; E′ and F′, Reelin; H′ and I′, p73) in *Ebf2*^*+/+*^ and *Ebf2*^*−/−*^ mice. Arrows point in Calr, Reelin and p73 samples to visible cells. Bar graphs show Calr, Reelin, p73 and PH3 cell counts at E13.5, E16.5 and E12.5, respectively (J–M). Layers in *Ebf2*^*−/−*^ mutants are correctly positioned, as confirmed by Nissl, Ctip2 and Cux1 staining (N–P). CH: cortical hem, MZ: marginal zone, Sp: septum. Scale bars: (B′–F′) 50 μm, (J–O) 100 μm.

The development of CR cells was analysed by tracking the number and position of CR cells, using CR subpopulation markers p73, Calr and Reelin. As *Ebf2* matched perfectly the expression of Reelin in the CH (Fig. 1F), but not in the septum and PCx (Figs. 1G–I), we focused our analysis only on CH-derived CR cells. Firstly, at E13.5, Calr^+^ cells, regardless of genotype, were found in the MZ and CH (Figs. 4A–C′, arrows). However, cell counts revealed a significant reduction in the number of Calr^+^ cells in the MZ of *Ebf2*^*−/−*^ mice compared to w-t controls (Fig. 4J; *Ebf2*^*+/+*^ 42 ± 2, *Ebf2*^*−/−*^ 31 ± 2; n = 6; p < 0.001). At the same time, more Calr^+^ cells were found in the CH of mutant animals, suggesting a possible problem in the migration of CR cells from this region into the cortex (Fig. 4J; *Ebf2*^*+/+*^ 44 ± 3, *Ebf2*^*−/−*^ 73 ± 3; n = 6; p < 0.001). Similarly, Reelin immunoreactive cell numbers (Figs. 4D–F′) were found to be significantly reduced in the MZ and increased in the CH of *Ebf2*^*−/−*^ mice (Fig. 4L; MZ, *Ebf2*^*+/+*^ 88 ± 2, *Ebf2*^*−/−*^ 79 ± 2; n = 3; p < 0.01; CH, *Ebf2*^*+/+*^ 68 ± 1, *Ebf2*^*−/−*^ 90 ± 1; n = 3; p < 0.05). Moreover, the number of p73 cells (Figs. 4G–I′), one of the best-described CR cell marker (Meyer et al., 2002, 2004; Tissir et al., 2009) was increased in the CH but not altered (Fig. 4K; MZ, *Ebf2*^*+/+*^ 55 ± 2.4, *Ebf2*^*−/−*^ 51 ± 1.4; n = 3; CH, *Ebf2*^*+/+*^ 64 ± 1.8, *Ebf2*^*−/−*^ 73 ± 2.1; n = 3; p < 0.01). Cajal–Retzius cell migration in pallial regions is terminated by E16.5 and, by this time, all such cells are positioned within the cortical MZ. Surprisingly, Calr- and Reelin-staining did not reveal any reduction in CR cell numbers in *Ebf2*^*−/−*^ animals at this stage (Figs. 4J′,L′; Calr, *Ebf2*^*+/+*^ 21 ± 3, *Ebf2*^*−/−*^ 23 ± 2; n = 3; Reelin, *Ebf2*^*+/+*^ 70 ± 3, *Ebf2*^*−/−*^ 65 ± 4; n = 3), indicating a transitory defect in the arrival of CR cells into the cortex. Intriguingly, we found a significant increase in cell proliferation in the CH, revealed by phospho-3-histone (PH3) immunoreactivity, in *Ebf2*^*−/−*^ mice compared to control littermates, only at E12.5 *(*Fig. 4M; pH3, *Ebf2*^*+/+*^ 50 ± 2, *Ebf2*^*−/−*^ 58 ± 2; n = 3).

**Supplementary Fig. 2:**
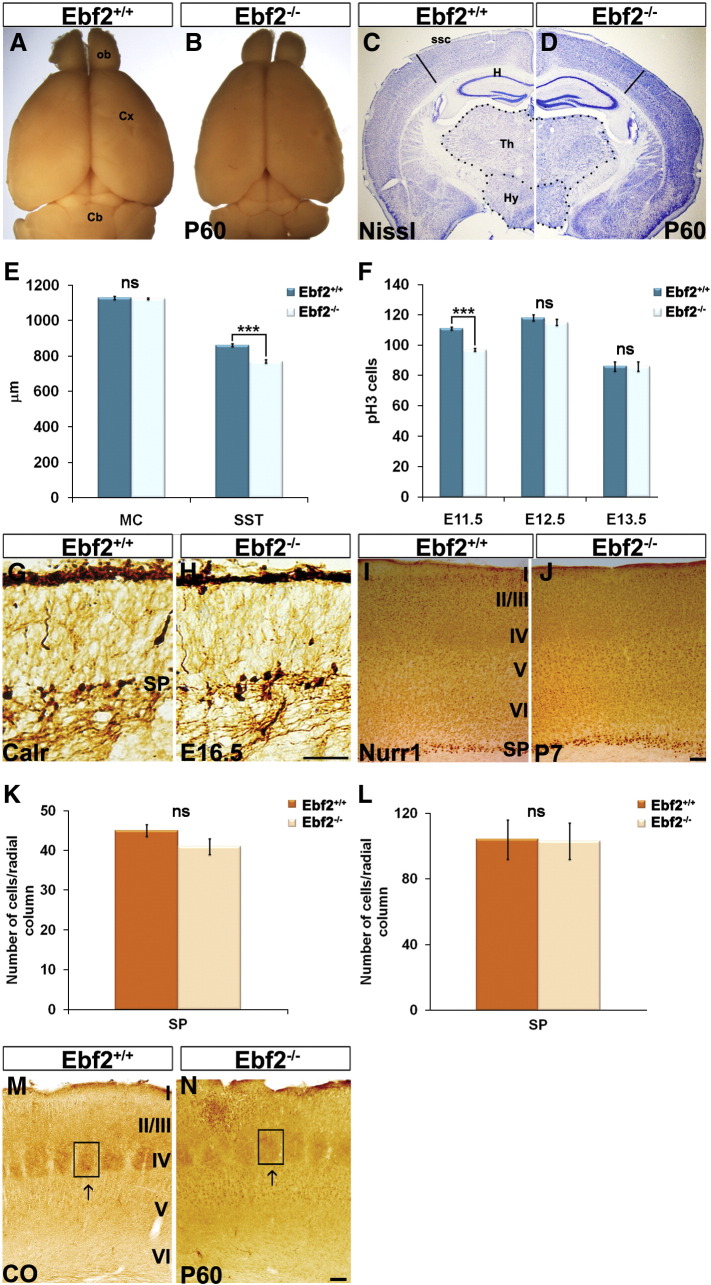
Analysis of the cortical thickness, SP cells and thalamocortical connections in *Ebf2*^*−/−*^ mice.

As *Ebf2*^*−/−*^ mice feature a transient delay in migration of CR cells at the early stages of development, we evaluated the thickness of the cerebral cortex and its layer organization postnatally. It is well established that loss of *Reelin* leads to disorganization of the cortical layers as seen in *Reeler* mutants (Caviness and Sidman, 1973). On the other hand, it has been shown that ablation of CH or CR cell loss does not necessarily lead to major cortical defects, suggesting that even a small amount of Reelin may be sufficient for correct cortical lamination (Rakic et al., 2006; Yoshida et al., 2006). Similarly, the six-layer organisation of the cerebral cortex, visualised by Nissl staining, was not affected in postanatal *Ebf2*^*−/−*^ mutants (Fig. 4N compared to N′). Moreover, immunostaining with specific markers for lower and upper cortical layers, Ctip2 and Cux1, respectively, demonstrated that these were not inverted in mutants compared to controls (Figs. O,P compared to O′, P′). However, we found an overall reduction in brain size in *Ebf2*^*−/−*^ mice (Figs. S2A,B), with significant thinning of the somatosensory cortex (SSC), but not motor cortex (MC) in *Ebf2*^*−/−*^ mutants compared to w-t controls (Figs. S2C,D; E:SSC, *Ebf2*^*+/+*^ 861 μm ± 8, *Ebf2*^*−/−*^ 770 μm ± 9; p < 0.001, n = 10; MC, *Ebf2*^*+/+*^ 1128 μm ± 9, *Ebf2*^*−/−*^ 1124 μm ± 4, n = 10). In line with this observation, we found a significant reduction in PH3^+^ cortical neuronal progenitors in *Ebf2*^*−/−*^ mutants compared to control littermates at a specific developmental age, E11.5 (Fig. S2F; E11.5: *Ebf2*^*+/+*^ 111 ± 1, *Ebf2*^*−/−*^ 97 ± 1; p < 0.05; E12.5: *Ebf2*^*+/+*^ 118 ± 2, *Ebf2*^*−/−*^ 115 ± 2; E13.5: *Ebf2*^*+/+*^ 86 ± 3, *Ebf2*^*−/−*^ 86 ± 3; n = 3). The observed reduction in cortical thickness prompted us to analyse the interneuron population to exclude defects in the *Ebf2* mutants, and found no significant changes in interneuron numbers in *Ebf2*^*−/−*^ mice compared to w-t controls (figures not shown; Calb, *Ebf2*^*+/+*^ 77 ± 2, *Ebf2*^*−/−*^ 82 ± 3; Calr, *Ebf2*^*+/+*^ 95 ± 3, *Ebf2*^*−/−*^ 89 ± 3; PV, *Ebf2*^*+/+*^ 231 ± 9, *Ebf2*^*−/−*^ 238 ± 10). We excluded thalamic and SP cell defects, by analysing the development of the SP layer in embryonic and postnatal animals (Figs. S2G–L) and barrel cortex (Figs. S2M,N). Furthermore, we analysed the integrity of the pyramidal cell population (Figs. 4O,O′: Ctip2, *Ebf2*^*+/+*^ 446 ± 22, *Ebf2*^*−/−*^ 412 ± 20; Figs. 4P,P′: Cux1, *Ebf2*^*+/+*^ 1474 ± 34, *Ebf2*^*−/−*^ 1346 ± 33; n = 3) and found a reduction in cell numbers that may explain the reduction in PH3 cells observed at E11.5 and the size of the adult cortex in the *Ebf2* mutants. These data suggest that *Ebf2* may affect the specification of particular pyramidal subpopulations (in layers IV and V).

In conclusion, it appears that *Ebf2* plays a pivotal role in the early migration of hem-derived CR cells. However, the rescue of the migratory defect at later stages of development and the absence of cortical lamination defect in adult mutants, suggest that this gene alone is not strictly necessary in regulating CR cell migration and laminar organization of the cortex.

### Ebf1 and Ebf3 gene expression overlap with Ebf2 during cortical development

**Fig. 5:**
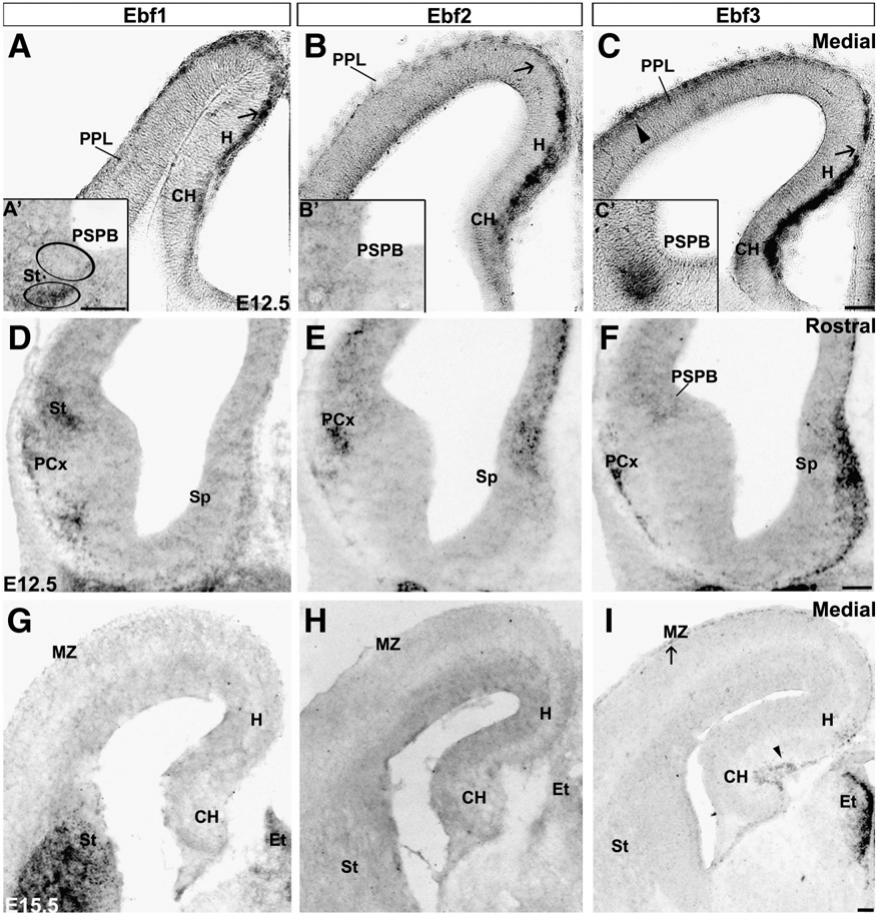
*Ebfs* expression in the developing forebrain. In situ hybridization shows that, at E12.5, *Ebf1, Ebf2* and *Ebf3* are expressed in similar territories (A–C). Arrows point to CH (A–C) and arrowhead to lateral PPL (C) indicating cells migrating from the CH and PSPB, respectively. *Ebf3* is detected in the PSPB (C′), whereas *Ebf1* in the striatum, but not in the PSPB (A′; circles delineate the St and PSPB). *Ebf2* and *Ebf3* are expressed in the septum (D–F). At E15.5, *Ebf1* and *Ebf2* are downregulated in the MZ and CH (G,H), whereas *Ebf3* is still detected in the CH, MZ and hippocampus (I; arrow and arrowhead, respectively). CH: cortical hem, PCx: piriform cortex, PPL: preplate, PSPB: pallial subpallial boundary, Sp: septum, St: striatum. Scale bars: (A–C; A′–C′) 100 μm, (G–I) 200 μm.

Previous studies have implied that *Ebfs* are expressed in overlapping territories of the developing forebrain and may have redundant roles (Garel et al., 1997). To identify the pattern of expression of *Ebf1* and *Ebf3*, forebrains of mice of different ages were analysed with in situ hybridization. *Ebf1* and *Ebf3* were detected from E10.5 to E15.5, when they were downregulated in a similar fashion to *Ebf2* (Figs. 1J–L). At E12.5, *Ebf3* expression was similar to that of *Ebf2* in the CH and PPL (Figs. 5B,C), however *Ebf3* was broadly expressed in the CH, whereas *Ebf2* was restricted to a subdomain (Fig. 5C compared to B). *Ebf1* was strongly expressed in the PPL, but was almost absent in the CH (Fig. 5A). Additionally, *Ebf3* was detected in the PSPB (Fig. 5C′). In the septum, *Ebf3* was expressed broadly along the midline, whereas *Ebf2* was confined dorsally (Figs. 5F,E), while no *Ebf1* expression was found (Fig. 5D). At E15.5, *Ebf1* was downregulated in the pallium (Fig. 5G), similar to *Ebf2* (Fig. 5H), but not in the striatum (Garel et al., 1997). *Ebf3* was still detected in the CH and MZ (Fig. 5I) and persisted in these areas after birth (data not shown). *Ebfs* are transiently expressed in the same forebrain structures, specifically in the CH and septum, and PPL/MZ, the sites of origin and migration of CR cells, respectively. Thus, these genes could act together to regulate CR cell development.

### The distribution of different CR cell subpopulations is affected in Ebf2^−/−^ mice

We then tested the hypothesis that the transient migratory defect seen in *Ebf2*^*−/−*^ mice may affect the distribution of the different CR subpopulations as previously reported by Griveau and colleagues (Griveau et al., 2010). The organization of the different CR subpopulations was analysed by in situ hybridization and immunohistochemistry for factors, including the *Ebf* genes, which are known to be specifically expressed in the septum, CH/PPL and PSPB during early development (E12.5).

**Fig. 6:**
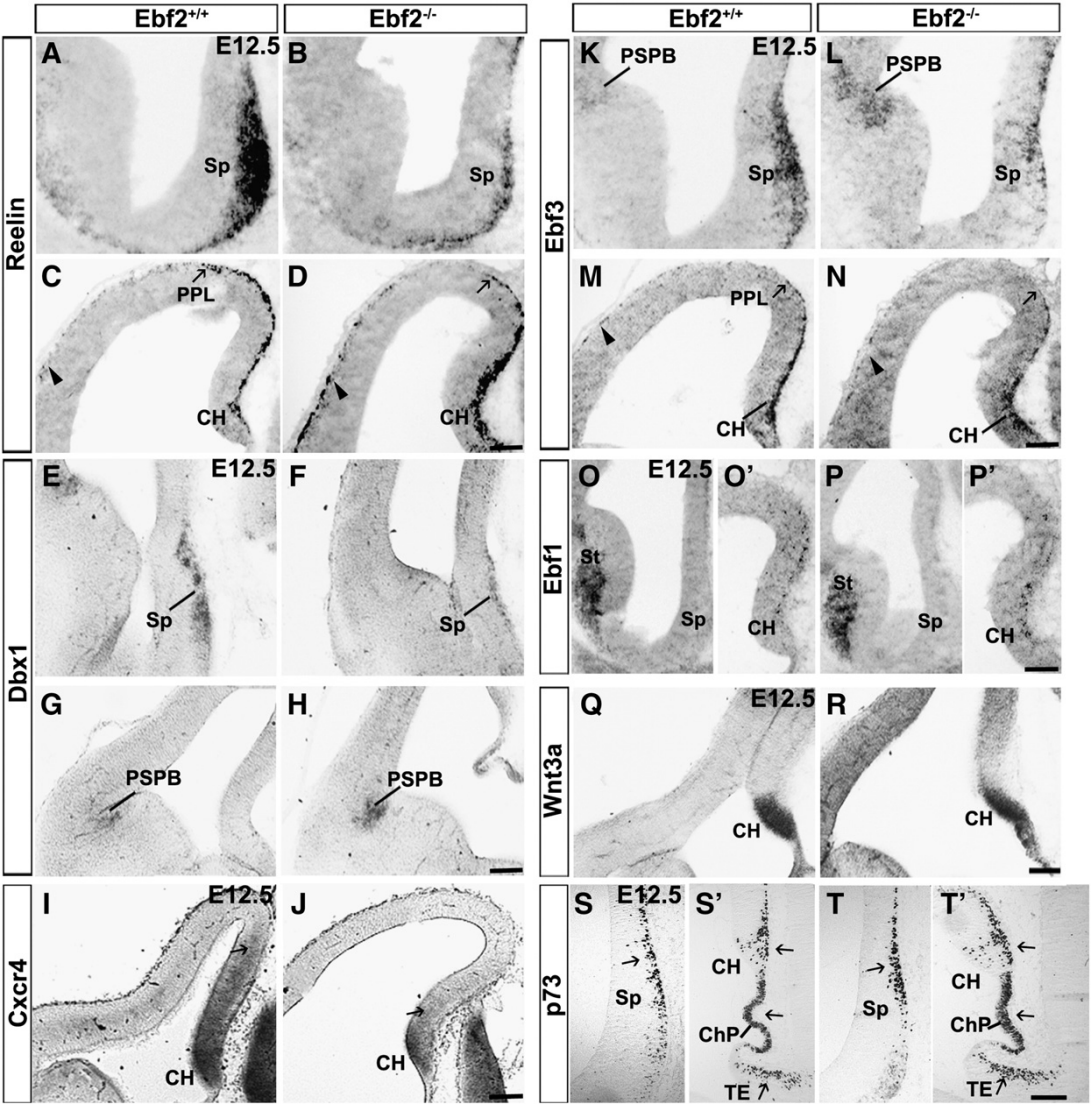
The distribution of CR cell subpopulations is affected in *Ebf2*^*−/−*^ mice. At E12.5, *Reelin* and *Dbx1* expressions are diminished in the septum and augmented in the CH and PSPB of *Ebf2*^*−/−*^ mice, respectively (A–H). *Cxcr4* expression was diminished in CH and in the dorsal pallium of *Ebf2*^*−/−*^ mice compared to w-t littermates (I, J). *Ebf3* in the CH and PSPB is extended in *Ebf2*^*−/−*^ mice compared to w-t controls, conversely it is diminished in the septum of *Ebf2*^*−/−*^ mice compared to w-t controls (K–N). Arrow shows that fewer cells migrate out the CH towards the dorsal PPL of *Ebf2*^*−/−*^ mice compared to w-t controls, whereas arrowhead points to cells in the lateral PPL, possibly deriving from the PSPB (A–N). *Ebf1* was unchanged (O–P′). *Wnt3a* domain in the CH is expanded in mutants compared to w-t controls (S, T). *p73* cells in the mutant septum are not dispersed along the medial axis as in w-t control (T compared to S, arrows). More p73cells are found in the mutant CH, ChP and TE (T′ compared to S′). CH: cortical hem, ChP: choroid plexus; PPL: preplate, PSPB: pallial subpallial boundary, Sp: septum, St: striatum, TE: thalamic eminences. Scale bars: 100 μm.

The expression of *Reelin* was found diminished in the septum of *Ebf2*^*−/−*^ mice compared to wt controls (Fig. 6A compared to B). Interestingly, a stronger signal for *Reelin* was detected in the CH of *Ebf2*^*−/−*^ mice compared to w-t controls (Fig. 6C compared to D). Furthermore, it was expressed less in the dorsal PPL and more strongly in the lateral PPL of *Ebf2*^*−/−*^ mice (Fig. 6D) compared to w-t controls (Fig. 6C), indicating that CR cells are trapped in the CH. *Dbx1*, a homeodomain transcription factor, specifies two CR cell subpopulations originating from the septum and PSPB (Bielle et al., 2005; Griveau et al., 2010). Its expression was found in the expected territories in *Ebf2*^*−/−*^ mice. However, *Dbx1* signal in the septum of *Ebf2*^*−/−*^ was reduced, whereas it was augmented in the PSPB compared to w-t controls (Fig. 6E compared to F, septum; G compared H, PSPB). *Ebf3* was expressed in the PPL (Fig. 6M) but, its expression was less in the dorsal PPL and more in the lateral PPL of *Ebf2*^*−/−*^ mice (Fig. 6N) compared to w-t controls. This phenotype together with the upregulation of *Ebf3* in the CH (Fig. 6M compared to N) resembled *Reelin* changes observed in *Ebf2*^*−/−*^ mice. Furthermore, similar to *Dbx1, Ebf3* expression was found diminished in the septum and increased in the PSPB in *Ebf2*^*−/−*^ mice compared to w-t controls (Fig. 6K compared to L). On the other hand, the expression of *Ebf1* in the CH was unchanged in *Ebf2*^*−/−*^ mice compared to w-t controls (Fig. 6O′ compared to P′)*. Ebf1* was not expressed in the septum of *Ebf2*^*−/−*^ animals as in w-t controls (Fig. 6O compared to P). *Wnt3a,* a secreted signalling molecule specifically expressed in the CH, was found in the expected territories of *Ebf2*^*−/−*^ mice, but expanded ventrally compared to wt controls (Fig. 6Q compared to R).

It has been reported that cortical meninges, expressing the chemokine *Cxcl12* (or *Sdf1*), act as a chemoattractant for CH-derived *Cxcr4*-expressing CR cells (Borrell and Marin, 2006). We found that *Cxcr4* signal was diminished and not dispersed dorsally in the CH of *Ebf2*^*−/−*^ mice (Fig. 6I compared to J, arrows), whereas the ligand *Sdf1* was expressed in expected territories in mutants and w-t controls (figures not shown). Additionally, p73 immunohistochemistry revealed an increased number of cells in the CH, ChP and thalamic eminences of *Ebf2*^*−/−*^ compared to w-t controls (Fig. 6S′ compared to T’; arrows). On the other hand, p73^+^ cells were localised in a single area of the septum of *Ebf2*^*−/−*^ (Fig. 6T′, arrow) compared to w-t p73 cells dispersed along the entire medial axis (Fig. 6T, arrow).

In summary, we observed the increased expression of *Reelin*, *Ebf3*, *Wnt3a* and p73 in the CH, as well as increased expression of *Dbx1* and *Ebf3* in the PSPB and p73 in the ChP and thalamic eminences. Conversely, we found reduced expression of *Dbx1* and *Ebf3* in the septum and *Cxcr4* in the CH. These changes together with the migratory defects observed in the *Ebf2*^*−/−*^ mutants strongly indicate that these genes, in particular *Ebf* factors, have compensatory roles during cortical development and may regulate CR cell motility. To test this hypothesis, we decided to analyse how overexpression and downregulation of single or multiple *Ebf* genes may affect neuronal migration.

### Ebf transcription factors control cell migration in a neuronal cell line

It has been shown that EBF proteins are critical for neuronal migration. For example, EBF factors regulate the migration of GnRH synthesizing neurons from the olfactory epithelium to the hypothalamus (Corradi et al., 2003), the migration of Purkinje neurons from the anterior cortical transitory zone to beneath the external granular layer in cerebellar cortex (Croci et al., 2006) and the migration of facial branchiomotor neurons in the hindbrain (Garel et al., 2000). Furthermore, when *Ebf1* is misexpressed in chick spinal cord, neuroepithelial progenitors migrate toward the mantle layer faster than normal, and the expression levels of *NF* and *R*-*cadherin* are upregulated (Garcia-Dominguez et al., 2003).

**Supplementary Fig. 3:**
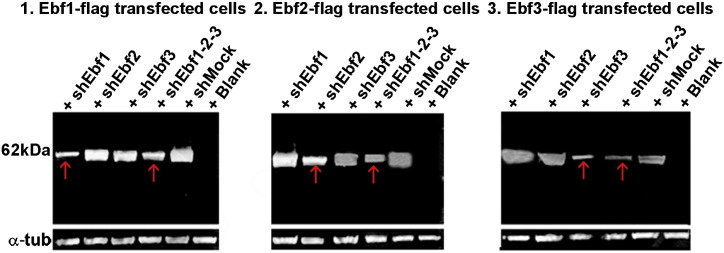
In vitro validation of overexpressing and silencing plasmids.

**Fig. 7:**
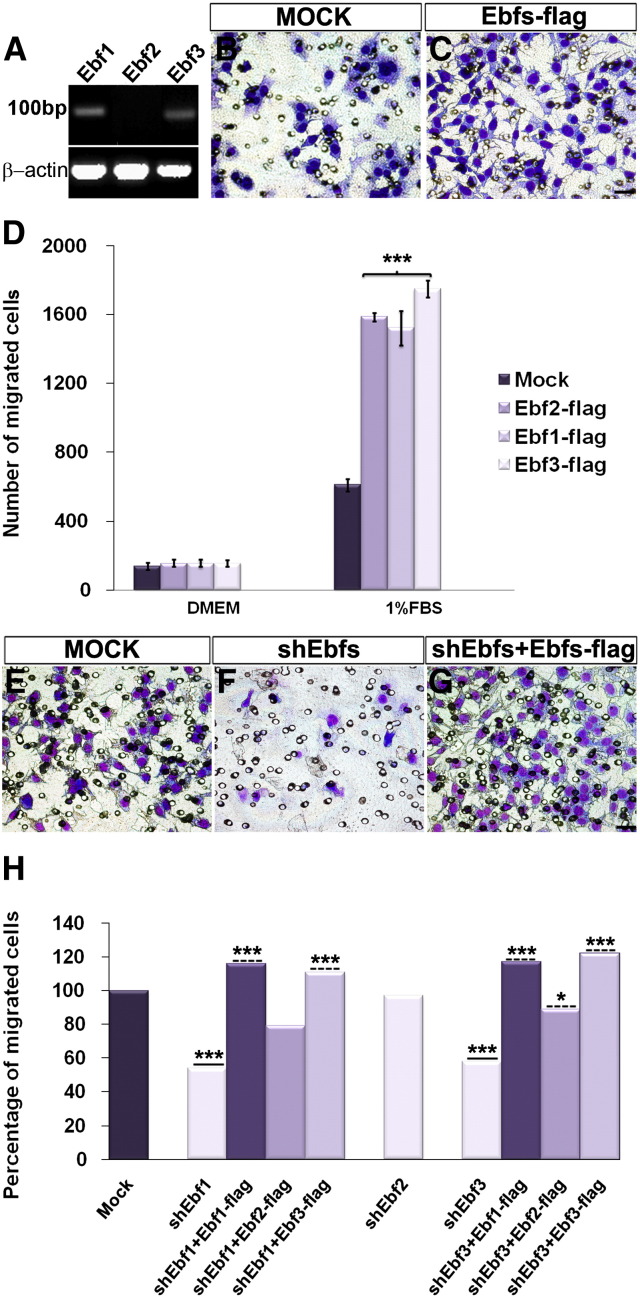
*Ebfs* overexpression and downregulation affect the migration of GN11 cells in vitro. *Ebf1* and *Ebf3* are expressed in GN11 cells (A). Fewer cells are visible in the *Mock* sample (B) compared to the *Ebf-flag* (C). Bar graph shows the increased migration of GN11 cells towards FBS when treated with *Ebfs* overexpression plasmids (D). GN11 cells do not migrate towards DMEM (D). Migration of GN11 cells when treated with *sh-Ebf1* and *Ebf3* is impaired compared to *Mock-* and *Ebf2-*treated (E–H). Migration is significantly rescued after transfecting with *Ebf-flag* plasmids *sh-Ebf* treated cells (G compared to F; H). Scale bar: 25 μm.

In line with this, we first used the GN11 cell line as a model to study migratory cell behaviour (Maggi et al., 2000) in response to the overexpression or downregulation of *Ebf* factors. GN11 cells naturally express *Ebf1* and *Ebf3,* but not *Ebf2* (Fig. 7A), thus not requiring further manipulation prior our experiments. The plasmids used were previously tested for specificity in COS cells (Figs. S3). GN11 cells, transfected either with *Ebf1*, *Ebf2* or *Ebf3*-*flag,* and exposed to a general chemoattractant (1% FBS) in a Boyden chamber assay, displayed a significant increase in chemomigration compared to mock-transfected cells (*Mock*) (Fig. 7B compared to C; D: *Mock* 609 ± 36, *Ebf1*-*flag* 1522 ± 30, *Ebf2*-*flag* 1585 ± 24, *Ebf3*-*flag* 1747 ± 48; n = 3; p < 0.001). Interestingly, *Ebf2*, even if not expressed by GN11 cells, was able, when overexpressed, to increase the rate of migration.

As overexpression of EBFs caused an increase in the chemomigration of GN11 cells, we subsequently analysed the effects of their downregulation on cell chemotaxis. Cells were transfected with *shEbf* plasmids and cultured for 72 h before the chemotactic assay. GN11 cells treated with *shEbf1* and *shEbf3* showed decreased migration compared to *Mock* and *shEbf2* treated GN11 cells (Fig. 7E compared to F; H: *Mock* 1273 ± 58, *shEbf2* 1200 ± 60, *shEbf1* 544 ± 59, *shEbf3* 603 ± 80; n = 3; p < 0.01). Interestingly, the migration of *shEbf1*-*2–3* transfected cells was more affected compared to GN11 cells treated with a single *shEbf*, suggesting that these factors together orchestrate cell migration and compensate each other's function (data not shown; *shEbf1*-*3* 500 ± 36; n = 3; p < 0.01). These results prompted us to test whether the defective migration of *shEbf* treated GN11 cells could be rescued by transfection with plasmids overexpressing the *Ebf* genes *(Ebf*-*flag)*. The defective migration of GN11 cells induced by *shEbfs* was indeed successfully and selectively rescued in cells overexpressing *Ebfs* (Fig. 7F compared to G; H: *Mock* 1273 ± 58; *shEbf1* 544 ± 59, *shEbf1* + *Ebf1*-*flag* 1172 ± 42; n = 3; p < 0.001; *shEbf3* 603 ± 80, *shEbf3 + Ebf3*-*flag* 1366 ± 57, n = 3; p < 0.001). *Ebf3*-*flag* rescued the migration of *shEbf1* treated cells (Fig. 7H: *shEbf1* 544 ± 59, *shEbf1* + *Ebf3*-*flag* 1299 ± 28, n = 3; p < 0.001), *Ebf1*-*flag* of *shEbf3* treated cells (Fig. 7H: *shEbf3* 603 ± 80, *shEbf3 + Ebf1*-*flag* 1325 ± 41, n = 3; p < 0.001) and, surprisingly, *Ebf2*-*flag* of *shEbf3* treated cells, but not *shEbf1* (Fig. 7H: *shEbf1* + *Ebf2*-*flag* 997 ± 36; *shEbf3* + *Ebf3*-*flag* 1366 ± 57, n = 3; p < 0.001).

We concluded that *Ebf* factors are strongly involved in neuron migration and compensate for each other's function in vitro. In particular, *Ebf2* is able to promote migration, even if not normally expressed by GN11 cells and, surprisingly, to compensate for the *Ebf3* loss of function. This data, in addition to the similarity of expression of *Ebf2* and *Ebf3* in the CH and the defects observed in the *Ebf2*^*/−*^ mice, suggest that EBF factors may cooperatively control migration of CH-derived CR cells.

### Redundant roles for Ebf2 and Ebf3 in motility of CH-derived CR cells

**Supplementary Fig. 4:**
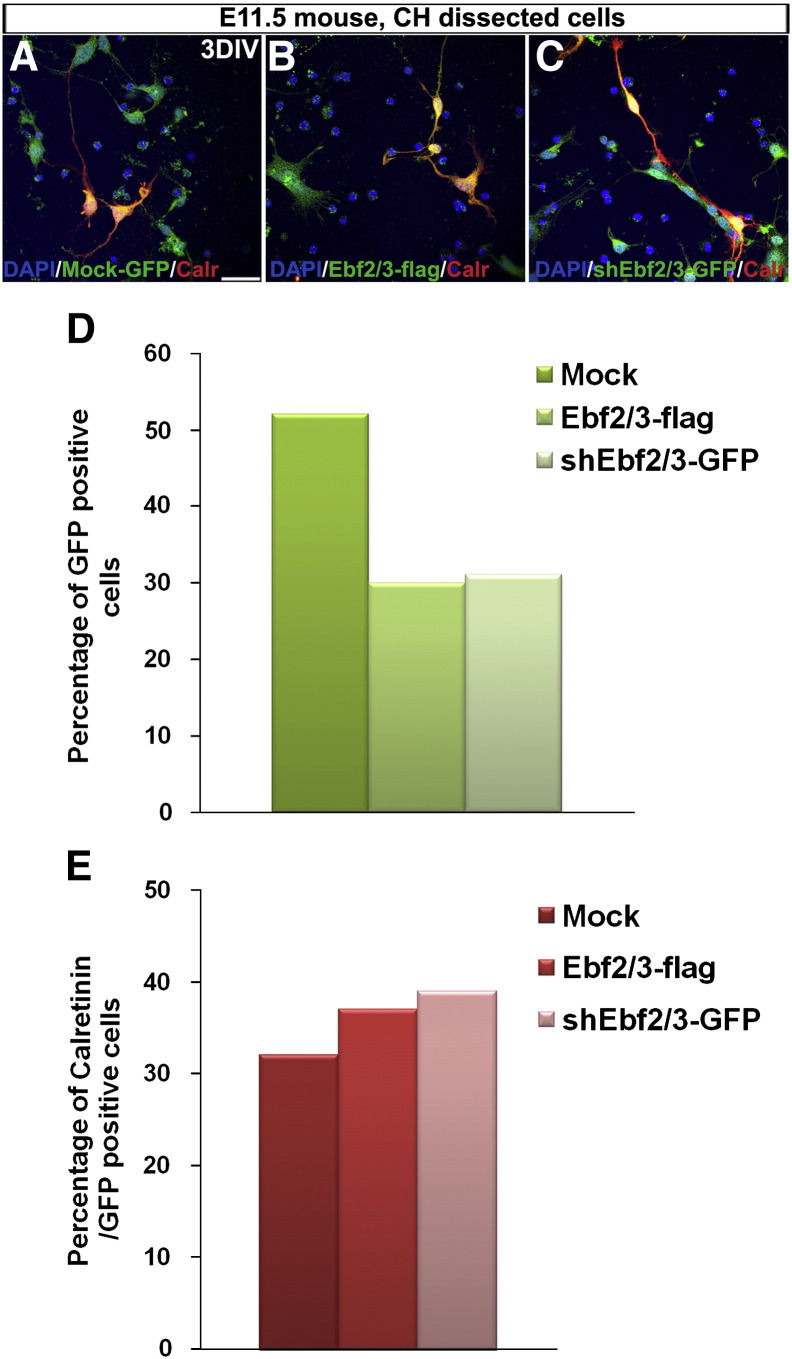
Validation of the stripe assay.

We next tested the hypothesis that *Ebf2* and *Ebf3* together play a role in the migration of CH-derived CR cells in vitro. We dissected cells from CH of E11.5 mice and electroporated them with *Ebf2/3*-*flag* or *shEbf2/3* plasmids. We then randomly plated cells in chambers containing poly-l-lysine (PLL) and laminin (LN) coated stripes to test their ability to move towards and adhere to LN (Halfter et al., 2002). The same amount of cells was used for each sample, and only transfected Calr^+^ cells, presumably CR cells, were considered (Figs. S4A–E).

**Fig. 8:**
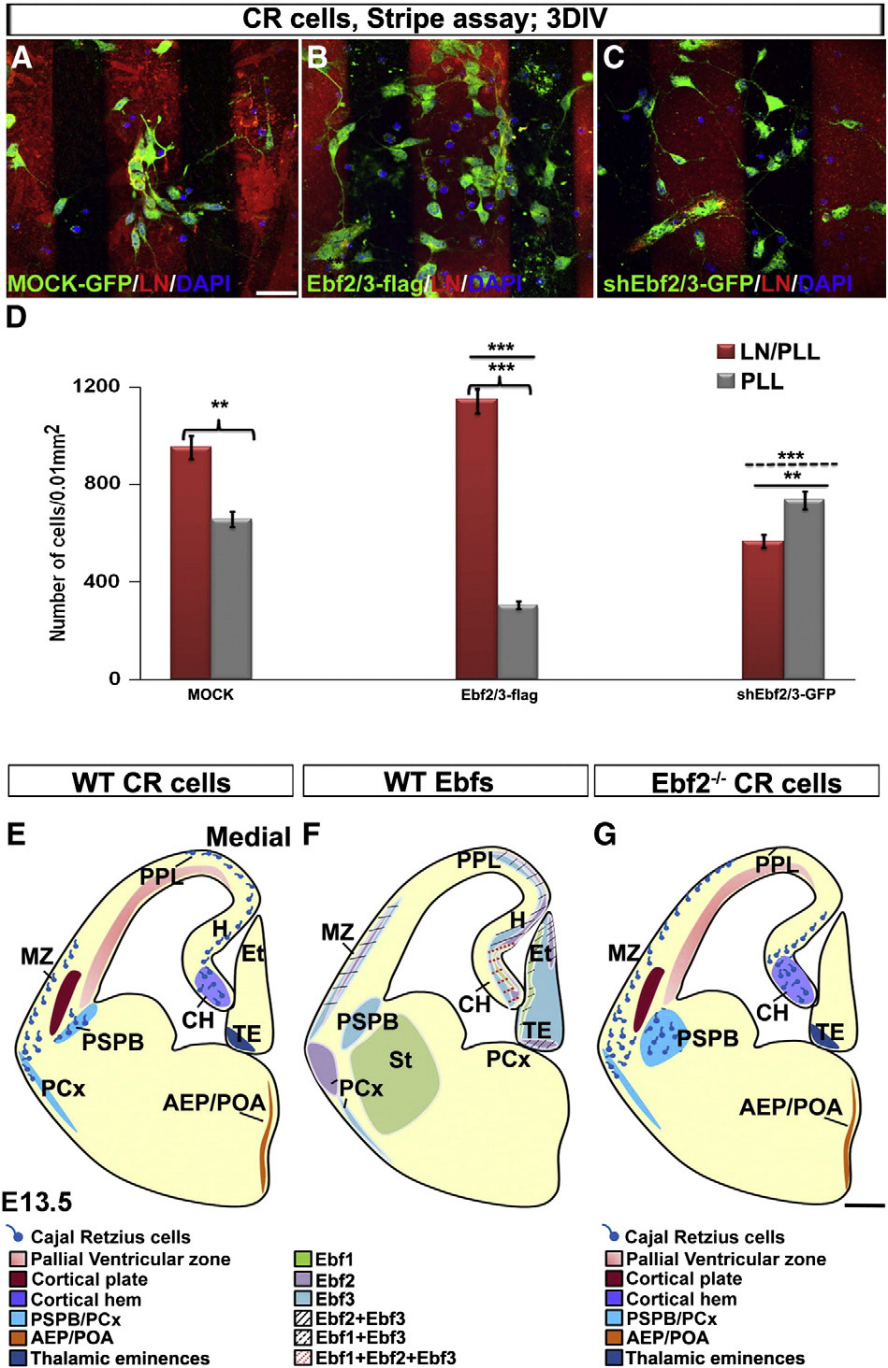
*Ebfs* overexpression and downregulation affect the migration of CR cells in vitro. *Mock*-treated cells prefer LN/PLL stripes compared to PLL only (*Mock*, A and D). *Ebf2/3-flag* overexpression increase the ability of CR cells to migrate towards LN stripes (B compared to A; D: *Ebf2/3*-*flag*, p < 0.001 LN/PLL compared to PLL; *Ebf2/3-flag* group compared to *Mock* group, p < 0.001, solid line). *Ebf2/3* downregulation decreases, instead, the ability of CR cells to migrate towards LN (C compared to A and B; D: *shEbf2/3-GFP* group compared to *Mock*, p < 0.01, solid line, or compared to *Ebf2/3-flag*, p < 0.001, dotted line). DIV: days in vitro, LN: laminin, PLL: poly-l-lysin. Scale bar: 25 μm.

The majority of *Mock* treated cells preferred PPL/LN to PLL stripes (Fig. 8A; D: LN/PLL 952 ± 79, PLL 657 ± 29; n = 3; p < 0.01). Similarly, cells overexpressing *Ebf2/3*-*flag* were mostly found on PPL/LN stripes (Fig. 8B compared to A; D: LN/PLL 1150 ± 82, PLL 305 ± 66; n = 3; p < 0.001), but in this case the amount of cells attached to LN/PLL was significantly higher when compared with the *Mock* treated samples, suggesting that they may require less time to migrate and attach to LN/PLL stripes (p < 0.001). Cells treated with *shEbf2/3* did not show any preference but, instead, they were randomly positioned in the chamber suggesting that they were unable to respond to the chemoattractant or requiring more time to reach the preferred stripe (Fig. 8C compared to A and B; D: LN/PLL 566 ± 88, PLL 735 ± 67; n = 3; p < 0.001 when compared to *Mock* and *Ebf2/3*-*flag*). Interestingly, cells transfected with single plasmids showed less severe phenotype (data not shown), indicating a redundant role for *Ebf2* and *Ebf3* in CH-derived CR cell migration.

## Discussion

In this study we examined the role of *Ebf* transcription factors in CR cell development. First, we identified *Ebf2* as a novel marker of CH- and septum-derived CR cells. Second, we described a redundant role for *Ebfs* in maintaining normal CR cell numbers and, consequently, proper lamination of the cerebral cortex. Finally, we recognised the importance of *Ebfs* in migration of CR cells arising in the CH.

### Ebf2 characterises CH- and septum-derived CR cells

In the telencephalon, *Ebf2* expression delineates the septum, CH, PPL/MZ (along the entire rostro-caudal axis) until E14.5, and its expression matches that of *Reelin*, a marker of CR cells. However, *Ebf2* and *Reelin* do not co-localise in the PCx or ChP, suggesting that *Ebf2* may have function(s) independent of CR development (Corradi et al., 2003). By using the mouse transgenic line *Ebf2*^*GFPiCre/R26R-YFP*^, we were able to effectively and permanently label *Ebf2*-expressing cells in the forebrain. More precisely, we have shown that *Ebf2* is expressed in CR cells that have originated from the CH and septum. Recent work by the Portera–Cailliau group (Chowdhury et al., 2010), using a similar transgenic line expressing GFP under the control of the *Ebf2* promoter, has shown that CR cells express GFP and, therefore, are EBF2^+^. However, this mouse line was used to study postnatal fate, but not the origin of CR cells.

### Specific roles for Ebfs in forebrain development

Here, we reported that embryonic *Ebf2*^*−/−*^ mice display a transient reduction in *Reelin*^+^ and Calr^+^ CR cell numbers in the PPL/MZ. However, these changes did not have any impact on PPL splitting (Figs. S2G,H) or cortical laminar organization (Figs. 4N–P′). The defects observed in *Ebf2*^*−/−*^ mice could be explained by the fact that *COE* genes encode phylogenetically conserved HLH transcription factors (Dubois et al., 1998; Pozzoli et al., 2001) expressed in overlapping territories in the developing head, including the olfactory epithelium, forebrain and cerebellum, and playing redundant as well as specific functions (Croci et al., 2006; Garel et al., 1997). We have shown that *Ebfs* are expressed in similar forebrain territories, in particular, within sites of origin and migration of CR cells. Regarding CR cell development, the differential expression of these genes suggests a possible mutual role for all three *Ebf*s in the CH/PPL, a common role for *Ebf2* and *Ebf3* in the septum, and exclusive role for *Ebf3* in the PSPB. These results link the *Ebf* transcription factors to CR cells, and imply that lack of cortical laminar defect in *Ebf2*^*−/−*^ mice may be due to a common function of *Ebf* genes in regulating the development of these cells.

Alternative compensatory mechanisms may be postulated to explain the rescue of *Ebf2*^*−/−*^ mouse cortical laminar phenotype. First, *Ebf2* loss of function is regained via CR cell redistribution, most likely from EBF2-negative regions, such as the signalling centre PSPB, ChP and thalamic eminences. Griveau et al. (2010) have previously shown that genetic ablation of the septum leads to a redistribution of PSPB- and CH-derived CR cells along with changes in early patterning that do not cause major cortical defects (Griveau et al., 2010). Accordingly, our findings strongly propose a redistribution of the CR subpopulations in *Ebf2*^*−/−*^ animals, as suggested by the expansion of the *Dbx1* and *Ebf3* domains in the PSPB. Alternatively, elevated expression of *Ebf3* and CH-specific marker *Wnt3a* together with increased proliferation, assessed by PH3 antibody in the CH of *Ebf2*^*−/−*^, suggest that *Ebf3* compensates for the loss of *Ebf2* in the CH. Furthermore, and in accord with our findings, *Reelin* and p73 were expressed more in the CH suggesting a delay in migration of CR cells. Intriguingly, less *Reelin/Calr* in the dorsal PPL, but increased signal in the lateral PPL/MZ were detected in *Ebf2*^*−/−*^ mice compared to w-t controls; however, the expression of p73 was unchanged in the PPL/MZ of mutants. This discrepancy may be explained by the presence of more p73 ChP- and thalamic-derived CR cells in the PPL/MZ of *Ebf* ^*−/−*^mice that have not yet started to express *Reelin* (Tissir et al., 2009).

Borrell and Marin (2006) have shown that the meningeal membranes are a necessary and sufficient substrate for the tangential migration of CH-derived CR cells in a *Cxcr4*-dependent manner. Our data suggest a delayed migration of CR cells that are retained longer in the CH of *Ebf2*^*−/−*^ mice. *Cxcr4* signal was diminished in the CH of *Ebf2*^*−/−*^ mice but, interestingly, the ligand *Sdf1* was normally expressed, suggesting the possibility that the chemokine receptor *Cxcr4* could be a downstream molecule of the *Ebf2* gene (Fig. 6). In addition, we observed that the expression of *Ebf3* and *Dbx1*, as well as *Reelin*, was reduced in the septum of *Ebf2*^*−/−*^ mice. As previously described, changes in the distribution of p73 cells affects the expression of *Reelin* (Meyer et al., 2004; Tissir et al., 2009). Thus, the defects observed in the localisation of p73 cells in the septum of mutants may explain changes in gene expression.

Finally, our expression data show that all CH-derived CR cells broadly express *Ebf3*, whereas *Ebf2* is found in a specific subpopulation and *Ebf1* only in migrating CR cells already in the PPL/MZ. In this model, *Ebf2* controls the early migration of a specific CR cell subpopulation by possibly affecting their ability to respond to *Sdf1*, later rescued by the redundant function of *Ebf3.* Croci et al. (2006) described a similar mechanism in the cerebellum, where *Ebf3* was expressed broadly in all Purkinje cells and *Ebf2* specifically in a subpopulation. Nevertheless, as also observed in the cerebellum (Croci et al., 2006), lack of *Ebf2* alone had an irreversible impact on brain size and thickness of the cerebral cortex, which could not be compensated by the presence of other EBF members. A plausible explanation for this phenotype is that absence of *Ebf2*-dependent CR cell population in the cortex of *Ebf2*^*−/−*^ mice, which carry crucial spatio-temporal cues relevant for proper cortical neurogenesis in a critical moment (E11.5; Fig. S2F), could result in permanent loss of cortical neurons and, consequently, reduction in thickness of the cortex. Additionally, *Ebf2* was expressed in pyramidal cells of layers IV–V, thus its loss of function may have affected their proliferation and specification, as suggested by changes in PH3, Ctip2 and Cux1 cell counts, respectively. However, further studies are required to elucidate a possible role of *Ebf* factors in pyramidal cell development.

### Importance of Ebf factors in CR cell migration

It has been shown that *Ebf* factors are strictly associated with cell migration in different species. *Ebf1* controls the expression of the adhesion molecule TAG1 in facial brachiomotor neuron migration (Garel et al., 2000), whereas *Ebf2* controls the migration of Purkinje cells in the cerebellum, and GnRH neurons from the olfactory epithelium to the hypothalamus through mechanisms that are still poorly understood (Corradi et al., 2003; Croci et al., 2006). We found that *Ebf2* and *Ebf3* play a compensatory role in the migration of CH-derived CR cells in vitro. This result was confirmed in a neuronal cell line (GN11 cells) that naturally expresses *Ebf1* and *Ebf3,* but not *Ebf2*. *Ebf2* overexpression caused an increase in cell migration, suggesting that this gene, even when not expressed, can trigger migratory mechanisms, most likely acting through the same signalling pathway as *Ebf3.* More significantly, *Ebf2* rescued *Ebf3* loss of function, indicating a synergistic action of these two *Ebfs* in cell migration. However, *Ebf2* did not rescue *Ebf1* loss of function indicating that these genes may be implicated in different migratory mechanism. Most likely, *Ebf1* is involved in the regulation of migration of CR cells in the PPL/MZ rather than in their initial exit from the CH. Moreover, stripe assay experiments showed that Calr^+^ cells overexpressing *Ebf2* and *Ebf3,* or both, were responding quicker to the chemoattractant laminin compared to w-t cells. The same cells transfected with siRNA against *Ebf2/Ebf3* were unable to migrate towards laminin and found scattered along the stripes. Interestingly, the downregulation of a single gene resulted in a less severe phenotype (data not shown). We also performed in utero and slice electroporation of the CH at E10.5 by injecting siRNA against *Ebfs* (data not shown). However, high embryonic mortality and the anatomical position of the CH made these experiments technically challenging and the results unreliable. However, future in vivo studies or analysis of double *Ebf2/3* mutants would help to clarify the role of these genes in the development of CR cells.

### Concluding remarks

As Reelin, secreted by CR cells, has been implicated in human psychiatric diseases such as schizophrenia (Frotscher, 2010; Ovadia and Shifman, 2011), it is particularly important to understand the role of genes involved in the production, migration and differentiation of these cells. We found that *Ebf* transcription factors are expressed in overlapping forebrain territories, and may have redundant roles in CR cell development. Although our findings offer a link between *Ebfs* and CR cells, future studies need to determine the downstream targets of these transcription factors in order to better understand corticogenesis and, possibly, the aetiology of psychiatric diseases.

The following are the supplementary materials related to this article.

Supplementary materials
