# Supplementary material for: Early B-cell factors 2 and 3 (EBF2/3) regulate early migration of Cajal–Retzius cells from the cortical hem
Source: Dev Biol. 2012 May 1;365(1-6):277–89. doi: 10.1016/j.ydbio.2012.02.034 (PMC3368273; doi:10.1016/j.ydbio.2012.02.034)
Supplement: Supplementary file 1 — Supplementary materials [file mmc1.doc]

Table S1 Primers for genotyping

| **MOUSE LINE** | **FORWARD (5’-3’)** | **REVERSE (5’-3’)** | **FRAGMENT SIZE (bp)** |
| --- | --- | --- | --- |
| ***Ebf2 ^-/-^*** | GAGGCGGCAGATCTGAAG | Wt-CCAATGCTGCCAGCAAATG  Mutant-CATTCAGGCTGCGCAACTGTT | Wt-250  Mutant-600 |
| ***Ebf2^GFPiCre^*** | ATGGTGCCAAGGATGACTCT | CCTCGAGCAGCCTCACCA | 250 |
| ***R26R^YFP^*** | Wt-GCGAAGAGTTTGTCCTCAACC  Mutant-AAAGTCGCTCTGAGTTGTTAT | GGAGCGGGAGAAATGGATATG | Wt-600  Mutant-250 |

Table S2 Primers for RT-PCR

| **GENE** | **PRIMER SEQUENCE (5’-3’)** | **FRAGMENT SIZE (bp)** | **SPECIES** |
| --- | --- | --- | --- |
| **m*Ebf1*-F** | AGGTTGGATTCTGCTACGAAAGTT |  | mouse |
| **m*Ebf1*-R** | TCAGGCCTTTTTAAGAGGAATCA | 80 |  |
| **m*Ebf2*-F** | TGGAGAATGACAAAGAGCAAG |  | mouse |
| **m*Ebf2*-R** | TTTGAAAACAGCGGGAAACCC | 330 |  |
| **m*Ebf3*-F** | TCGTGAATATGCACCGTTTTG |  | mouse |
| **m*Ebf3*-R** | CCTCGAGACATTTTTTCTGTACTCAT | 76 |  |

Table S3 Plasmids for cell transfection

| **NAME** | **5’ TAG** | **PLASMID** |
| --- | --- | --- |
| ***Ebf1-flag*** | FLAG | pcDNA 3.1 |
| ***Ebf2-flag*** | FLAG | pcDNA 3.1 |
| ***Ebf3-flag*** | FLAG | pcDNA 3.1 |
| ***shEbf1*** | GFP | pcDNA 6.2 |
| ***shEbf2*** | GFP | pcDNA 6.2 |
| ***shEbf3*** | GFP | pcDNA 6.2 |
| ***shEbf1-2-3*** | GFP | pcDNA 6.2 |
| ***shMock*(scrambledRNA*)*** | GFP | pcDNA 6.2 |

Table S4 Primary antibodies

| **ANTIGEN** | **HOST** | **CLONE** | **DILUTION** | **SUPPLIER** |
| --- | --- | --- | --- | --- |
| **Ctip2** | rat | Polyclonal | 1/1000^H^ | Abcam |
| **GFP** | rabbit | Polyclonal | 1/3000^H^ | Abcam |
| **GFP** | chicken | Polyclonal | 1/500^H^ | Aves |
| **pan-Ebf** | rabbit | polyclonal | 1/2000^WB^ | Sigma |
| **Reelin** | mouse | Monoclonal | 1/3000^H^ | Gift of F. Goffinet |
| **Nurr1** | rat | Polyclonal | 1/100^H^ | Z. Molnar |
| **PH3** | rabbit | Polyclonal | 1/100^H^ | Abcam |
| **Cux1** | rabbit | Polyclonal | 1/100^H^ | Santa Cruz |
| **a-tubulin** | mouse | Monoclonal | 1/5000^WB^ | Sigma |
| **Paravalbumin** | mouse | Monoclonal | 1/500^H^ | Sigma |
| **FLAG** | mouse | Monoclonal | 1/100^H^ | Sigma |
| **Calbindin** | Rabbit | Polyclonal | 1/5000^H^ | Swant |
| **Calretinin** | Rabbit | Polyclonal | 1/2000^H^ | Swant |

H: Immunohistochemistry, WB: Western blot.Abcam, Cambridge, UK; Aves Labs, Oregon, USA; RnD Systems, Abingdon, UK; Santa Cruz, Heidelberg, Germany; Swant, Bellinzona, Switzerland; F. Goffinet, Catholic University of Leuven, Leuven, Belgium

Table S5 Secondary antibodies

| **ANTIBODY** | **HOST** | **CONJUGATED WITH** | **DILUTION** | **SUPPLIER** |
| --- | --- | --- | --- | --- |
| **Anti-mouse IgG** | goat | Biotin | 1/200 | Vector |
| **Anti-rabbit IgG** | goat | Biotin | 1/200 | Vector |
| **Anti-rat IgG** | goat | Biotin | 1/200 | Vector |
| **Anti-goat IgG** | donkey | Biotin | 1/200 | Vector |
| **Anti-chicken IgG** | goat | FITC | 1/1000 | Invitrogen |
| **Anti-mouse IgG** | goat | FITC | 1/1000 | Invitrogen |
| **Anti-rabbit IgG** | goat | FITC | 1/1000 | Invitrogen |
| **Anti-rat IgG** | goat | FITC | 1/1000 | Invitrogen |
| **Anti-rat IgG** | donkey | TRITC | 1/1000 | Invitrogen |
| **Anti-mouse IgG** | goat | TRITC | 1/1000 | Invitrogen |
| **Anti-rabbit IgG** | goat | TRITC | 1/1000 | Invitrogen |
| **Anti-mouse IgG** | goat | HRP | 1/5000 | Amersham |
| **Anti-rabbit IgG** | goat | HRP | 1/5000 | Amersham |

FITC: fluorescin-5-isothiocyanate (Alexa Fluor 488), TRITC: tetramethilrhodamine-5-(and 6)- isothiocyanate (Alexa Fluor 568), HRP: horseradish peroxidase. Vector, Peterborough, UK; Amersham, GE Healthcare, Netherlands

Supplementary Fig. 1. Validation of the *Ebf2^GFPiCre^* transgenic mouse.

This transgenic line was obtained through the integration of a GFP protein fused with an improved Crerecombinase protein (*GFPiCre)* construct (Badaloni A, unpublished) targeted into the first coding exon of the *Ebf2* locus in the BAC RP24-283N8 (A). This BAC contains the *Ebf2* promoter, the beginning of the *Ebf2* coding sequence located in exon 2 and the first seven exons of the gene (for BAC specifications: Gene Expression Nervous System Atlas, GENSAT, www.gensat.org). The transgene*GFPiCre* was inserted through bacterial recombination in the second exon as shown in (A). At E13.5, *GFPiCre* expression matched the expression of *Ebf2* in the CH, septum, hippocampus, cortical PPL/MZ, SP and PCx (B, C). At E15.5, dowregulation of transgene signal occurred in the expected territories such as CH, septum, hippocampus and MZ, but persisted in the PCx (D, E). LoxP recombination driven by the Crerecombinase at the *Rosa26* locus leads to the activation of the Crerecombinase, the excision of the stop signal and the permanent expression of the YFP protein in *Ebf2* expressing cells (F).Whole-mount YFP expression in *Ebf2^GFPiCre/R26R-YFP^* at E13.5 and E17.5 shows the activity of Crerecombinase in *Ebf2^+^* cells, allowing *GFPiCre*-expressing cells and their progeny to be indelibly labelled *in vivo*, even long after the *Ebf2* gene is developmentally downregulated (G, H). CH: cortical hem, Cb: cerebellum, F: forebrain, H: hippocampus, HB: hindbrain, HOM: homology arms for recombination, MB: midbrain, MZ: marginal zone, OB: olfactory bulb, oe: olfactory epithelium, PA: polyadenilation signal, PCx: pyriform cortex, PPL: preplate, SP: subplate, YFP: yellow fluorescent protein.Scale bars: (B-E) 100 µm, (G, H) 50 µm.

### Supplementary Fig. 2. Analysis of the cortical thickness, SP cells and thalamocortical connections in *Ebf2^-/-^* mice.

The size of the cerebral hemisphere was visibly reduced in *Ebf2^-/-^* mice compared to w-t controls (A,B). Moreover the somatosensory cortex (SSC), but not motor cortex (MC), was significantly thinner in *Ebf2^-/-^* mutants compared to w-t controls (C,D; E:SSC, *Ebf2^+/+^* 861 μm±8, *Ebf2^-/-^*770 μm ±9; p<0.001, n=10;MC, *Ebf2^+/+^* 1128 μm±9, *Ebf2^-/-^*1124 μm±4, n=10;). We found a significant reduction in PH3^+^ cortical neuronal progenitors in *Ebf2^-/-^* mutants compared to control littermates at a specific developmental age, E11.5 (Fig. S2F; E11.5: *Ebf2^+/+^* 111±1, *Ebf2^-/-^*97±1; p<0.05; E12.5: *Ebf2^+/+^* 118±2, *Ebf2^-/-^*115±2; E13.5: *Ebf2^+/+^* 86±3, *Ebf2^-/-^*86±3; n=3). At E16.5, the SP layer is fully developed, and cells are clearly visible and labelled with Calr (G,H). There are no significant changes detected in the cell number in *Ebf2^-/-^* mice compared to w-t littermate controls (K:*Ebf2^+/+^* 45±3, *Ebf2^-/-^* 41±2; n=3). Postnatally, the number of Nurr1^+^ cells (I,J) is counted in mutants and compared to w-t animals. Changes in position or cell number were not detected in *Ebf2^-/-^* mice (L:*Ebf2^+/+^* 104±12, *Ebf2^-/-^* 103±11; n=3).

In order to exclude thalamic defects, suggested also by the thalamic area reduction seen in mutants (data not shown), the correct formation of the barrel cortex was analysed. The barrel cortex refers to dark-stained regions found in layer IV of the SSC that receive inputs from the thalamus. At P7, barrels were visualized CO staining in mutants and control animals (M,N; squares and arrows delineate the barrels) and they are organised with the expected structure (the septa are clearly visible between barrels). Calr: calretinin, Cb: cerebellum, CO: cytochrome oxydase, Cx: cortex, H: hippocampus, Hy: hypothalamus, Nurr1: nuclear related receptor 1, ob: olfactory bulbs, SP: subplate, ssc: somatosensory cortex, Th: thalamus. Scale bar: (G,H)150 µm, (C,D; I, J; M, N) 100 µm.

### Supplemetary Fig. 3. In vitro validation of overexpressing and silencing plasmids.

The specificity of the *Ebf-flag* and *short hairpin (sh)Ebf* plasmids were tested *in vitro* in COS cells, which do not express any of the COE genes (data not shown). Confluent COS cells were transfected with *Ebf1/2/3-flag* separately and cultured for 24 h. Cells were split and subsequently transfected with different combinations of *shEbfs* as follows:

*3. Ebf3-flag +*

*a) shEbf1*

*b) shEbf2*

*c) shEbf3*

*d) shEbf1-2-3*

*e) shMock*

*2. Ebf2-flag +*

*a) shEbf1*

*b) shEbf2*

*c) shEbf3*

*d) shEbf1-2-3*

*e) shMock*

*1. Ebf1-flag +*

*a) shEbf1*

*b) shEbf2*

*c) shEbf3*

*d) shEbf1-2-3*

*e) shMock*

Non transfected COS cells were used as negative controls and indicated as Blank. After 72 h past the second transfection with the *shEbfs*, cells were collected and proteins extracted to be used for Western blot analysis. Proteins were quantified, and the same amount was used for each sample (α-tubulin shows protein concentration). The intensity of the *shMock* (containing scattered RNA, used as positive control) band on the blot was used as reference. A significant reduction in the expression of the EBF proteins compared to *shMock* transfected cells was observed, as expected, in the following samples (red arrows):

- *Ebf1-flag*transfected COS cells and treated with *shEbf1* and *shEbf1*-*2-3*
- *Ebf2-flag*transfected COS cells and treated with *shEbf2* and *shEbf1*-*2-3*
- *Ebf3-flag*transfected COS cells and treated with *shEbf3* and *shEbf1-2-3*

α-tub: alpha-tubulin, kDa: kilo-Dalton

### Supplemetary Fig. 4. Validation of the stripe assay.

### CH-derived CR cells are dissociated from E11.5 mouse, transfected and cultured for 3 days in vitro to check the efficiency of transfection and Calr expression as a specific CR cell marker (A-C). The rate of transfection was 50% for Mock to 30% for *flag* and *sh-Ebf* treated CR cells (D). The rate of expression of Calr among the transfected cells was between 30-40% (E). CR cells, independently by the plasmids they were transfected with, show clear visible projections (A-C). CH: cortical hem, Calr: calretinin, DAPI: 4',6-diamidino-2-phenylindole. Scale bar: 50 µm.
